# An Interesting Case of Obstetrics, with General Remarks

**Published:** 1877-10

**Authors:** 


An Interesting Case of Obstetrics, with General Remarks. On the morning of August 18th, 1877, I was called in consultation by a partner, Dr. J. K. Stone, to render him assistance in a case of labor—primipara. On digital examination, I found that I could penetrate but about an inch into the vagina. I recognized a tense, unyielding membrane. Ocular examination revealed a tense muscular septum occluding the vagina, there being only a small orifice at the urethral portion, through which I could pass a small probe, and through which, with each pain was passing a little bloody fluid. I informed husband and parents and asked for consultation, for I feared, besides the vaginal trouble, there might be deficiency in the bony pelvis, as externally she was very ill-formed, partaking more of the masculine physique. Dr. Tottenham, of Sempronius, answered my call, and in ten hours from the time I first saw her we attempted delivery. Dr. J. K. Stone administering chloroform, and Dr. Tottenham, with his fingers, holding open the vulva and vagina, I passed a small probe through the orifice, and with the probe as a guide I punctured with a curved bistoury, and then inserted the probe-pointed bistoury, and laid open the obstruction antero-posteriorly to more than an inch in extent. Expulsive pains coming on did the rest. Now having room, I at once applied Brickell's forceps without any trouble, and with the next contraction of the uterus succeeded in effecting the delivery of a nine-pound child.

Dr. Tottenham and myself having been pupils of Professor Brickell, adopted his plan of immediate delivery of the placenta. The uterus at first contracted tolerably well, but in a few seconds there occurred alarming hemorrhage. Dr. Stone administered sixty minims of Stark and Dohme's liquor ergotæ hypodermically, Dr. Tottenham applied cold, and I injected solution perchloride of iron into the uterus. We succeeded in checking the hemorrhage, and in one hour from the commencement of the operation all was well. We washed out the vagina for ten days, following the delivery with a carbolic acid solution. There was no fever, or any untoward trouble, and now the organs are entirely well. The membrane was not allied to the hymen, being musculo-fibrous, and the husband and wife assured me that until within the month previous to delivery there had been no obstacle to coition. The tissue at the point of junction with the urethra was a quarter of an inch thick, very vascular.

The woman had never had any trouble in menstruation, though on account of a scrofulous vitiation and anæmia, she was rather late commencing.

One further remark. When a student of medicine under D. W. Brickwell, we were always taught that after the delivery of the child the placenta was to be speedily delivered, it being now a foreign body *in utero*. His plan I have adopted in practice, and I have never yet had any trouble result, although I have been called in on two occasions to deliʌer thə placenta after fourteen and twenty-four hours, by competent and skilled physicians, who had been taught otherwise, and had strong faith in the *vis medicatrix naturæ*. In the fourteen hour case there were no ill effects; in the twenty-four hour case the placenta was putrid, and the woman had puerperal peritonitis with partial sloughing of the vagina. With one hand to make gentle traction on the cord and the other to knead the uterus, I cannot think there can be any danger of producing inversion. I make these remarks because I notice a discussion going on in the journals, in regard to the management of the third stage of labor.

To another point my attention has been called by a late article in the *Obstetrical Journal of Great Britain and Ireland*, viz: the effects of narcotics and hypnotics on the fœtus. I have noticed that in labor without these agents, although the time consumed might be the same, there was less difficulty in resuscitating the child, and in one case where we had to keep the woman under prolonged narcotism with morphia and chloroform, the child showed the effects of the agents and lived *but a short time*—and it was an entirely different condition from the asphyxia of Butt.—*New Orleans Medical and Surgical Journal.*

